# Non surgical predicting factors for patient satisfaction after third molar surgery

**DOI:** 10.4317/medoral.20719

**Published:** 2016-01-31

**Authors:** José-Carlos Balaguer-Martí, Amparo Aloy-Prósper, David Peñarrocha-Oltra, Miguel Peñarrocha-Diago

**Affiliations:** 1 Master of Oral Surgery and Implantology. Valencia University Medical and Dental School; 2 Collaborating Professor of Oral Surgery. Master of Oral Surgery and Implantology. Valencia University Medical and Dental School, Valencia, Spain; 3 Professor of Oral Surgery. Director of the Master of Oral Surgery and Implantology. Valencia University Medical and Dental School

## Abstract

**Background:**

In the third molar surgery, it is important to focus not only on surgical skills, but also on patient satisfaction. Classically studies have been focused on surgery and surgeon’s empathy, but there are non-surgical factors that may influence patient satisfaction.

**Material and Methods:**

A cross-sectional study was performed on 100 patients undergoing surgical extractions of impacted mandibular third molars treated from October 2013 to July 2014 in the Oral Surgery Unit of the University of Valencia. A questionnaire (20 questions) with a 10-point Likert scale was provided. The questionnaire assessed the ease to find the center, the ease to get oriented within the center, the burocratic procedures, the time from the first visit to the date of surgical intervention, waiting time in the waiting room, the comfort at the waiting room, the administrative staff (kindness and efficiency to solve formalities), medical staff (kindness, efficiency, reliability, dedication), personal data care, clarity in the information received (about the surgery, postoperative care and resolution of the doubts), available means and state of facilities. Outcome variables were overall satisfaction, and recommendation of the center. Statistical analysis was made using the multiple linear regression analysis.

**Results:**

Significant correlations were found between all variables and overall satisfaction. The multiple regression model showed that the efficiency of the surgeon and the clarity of the information were statistically significant to overall satisfaction and recommendation of the center. The kindness of the administrative staff, available means, the state of facilities and the comfort at the waiting room were statistically significant to the recommendation of the center.

**Conclusions:**

Patient satisfaction directly depends on the efficiency of the surgeon and clarity of the clinical information received about the procedure. Appreciation of these predictive factors may help clinicians to provide optimal care for impacted third molar surgery patients.

**Key words:**Patient satisfaction, third molar, questionnaire.

## Introduction

In the field of oral and maxillofacial surgery, impacted third molar surgery (ITM) is one of the most common procedures (1). ITM surgery appears to be a relatively minor operation with few complications and little morbidity. However, ITM surgery is often perceived by patients as an intensely frightening situation (2,3). Likewise, a patient is satisfied when a surgery performs better than expected and is dissatisfied when expectations exceed performance. Favorable outcomes leads to patient satisfaction (4,5). However, the relationship between surgeon and patient may be endangered by environmental factors not only surgical outcomes (1,2).

Scher *et al*. (6) highlighted the importance of patient satisfaction within the basic principles in measuring quality. Satisfaction surveys are ways in which the patient is asked about their satisfaction on the health care provided. Moreover, the factors or causes that may influence the level of satisfaction, such as accessibility, technical competence of the professionals, the interpersonal relationships and humane treatment, and cleanliness must be considered. Badia *et al*. (7) conducted a study in 1998 on the aspects of dental care that are most valued by patients, and it was determined that not all factors are valued in the same way. The most important component for the patient satisfaction reported by the literature was the effectiveness of the surgeon, including technical skills and confidence (8), but there is a lack of evidence for the non-surgical factors related with the ITM surgery.

Appreciation of the factors increasing patient satisfaction may guide clinicians to provide optimal care for their patients. Most clinicians, including oral and maxillofacial surgeons, focus only on a desirable surgical outcome when they treat ITM patients. However, a large gap exists between the provision of ideal care and patient recognition of ideal care, because most patients do not have sufficient knowledge to evaluate surgical outcomes. As the patient determines whether a service is acceptable or not, the clinician should know what patients need and prefer, to design and improve the assistance. For this reason, the aim of the present study was to determine significant factors predicting patient satisfaction regarding the center and care provided by practitioners and administrative staff after third molar surgery.

## Material and Methods

- Sample selection

An observational cross-sectional study was performed following the STROBE guidelines (9) including 127 patients that underwent surgical extraction of an impacted mandibular third molar (totally covered by bone, totally covered by soft tissues or partially covered by soft tissues) in the Oral Surgery Unit of the University of Valencia from November 2013 to July 2014. The study was approved by the Ethics Committee of the University of Valencia (H1435828552407). The inclusion criteria were healthy patients, older than 18 years, who completed the questionnaire and agreed to follow the postoperative instructions. All patients signed an informed consent to be included in the study.

A questionnaire was prepared using a Likert-type scale, consisting of a set of 20 items rated from 1 to 10 (1, strongly disagree; 10, strongly agree) to assess the patient satisfaction related to all questions. The questionnaire assessed the ease to find the center, the ease to get oriented within the center, the burocratic procedures performed during the first day arriving at the center, the time from the first visit to the date of surgical intervention, waiting time in the waiting room the day of the intervention, the comfort at the waiting room, the administrative staff (kindness and efficiency to solve formalities), medical staff (kindness, efficiency, reliability, dedication), personal data protection, clarity in the information received (about the surgery, postoperative care and resolution of the doubts), available means and state of facilities (cleanliness, performance...). These items were selected and included in our questionnaire from different surveys that assessed the patient satisfaction with the Spanish National Health System. The questionnaire was provided to the patient one week after the ITM surgery. The meaning of the questions and the criteria of rating was explained. Subsequently, they were asked to fill in the questionnaire and instructed to give the most accurate rating to the prescribed set of questions. Furthermore, they were instructed to fill out the questionnaire in isolation of the operator or other relatives to avoid any bias in rating the answers to the questions. The patients were instructed to abstain from writing their names or putting their signatures on the questionnaire forms in an effort to protect their identity. The filled questionnaire was collected by the administrative staff, different from the researcher.

- Outcome measures

Outcome variables to assess patient satisfaction were: the overall satisfaction (SP) and the recommendation of the center (ROC).

- Surgical procedure

All surgeries were performed using identical surgical instruments and material by one surgeon. The patients were referred from the Spanish National Health System. In all cases, the inferior alveolar, lingual and buccal nerves were anesthetized using 2 cartridges of 1.8 ml of articaine 4% and epinephrine anesthetic solution at 1:100000 Artinibsa® (Inibsa, Lliça de Vall, Barcelona, Spain). A vestibular triangular mucoperiosteal flap was raised with a distal incision and vestibular release. The osteotomy and odontectomy were made using a rounded tungsten carbide drill, mounted in a hand piece, with abundant irrigation of sterile physiologic serum. After extracting the molar, the cavity was inspected and sutured with 3-0 silk (Lorca Marin, TB15, 3/8, Murcia, Spain). All patients were prescribed the same prophylactic antibiotic (500mg amoxicillin orally) one tablet every eight hours for one week, and ibuprofen (Bexistar, Laboratory, Bacino, Barcelona, Spain) 600 mg every 8 hours for 4 days. The patients were advised to consume a soft diet for the first 24 hours and to abstain from smoking during the first postoperative week. Brushing at the surgical site was limited to the occlusal or incisal surfaces of the teeth, with careful brushing of all other teeth (brushing 3 times daily). Sutures were removed one week after the surgery.

- Statistical analysis

The collected data was tabulated and statistically evaluated. Frequency distribution and percentage analysis were done. The internal consistency of the items included in the questionnaire was analyzed with the Cronbach’s alpha to ensure that all the items measured the same outcome variable (satisfaction). Spearman’s correlation coefficient was used for correlating the individual experience with the OS and ROC variables. Linear regression was used to find correlations between the studied variables and the outcome variables OS and ROC. The Cook’s distance was used to assess if the atypical values influence the fit of the regression model. Stratification was performed at the age variable to compare data between ranges of twenty years. The level of statistical significance was established as 5% (a = 0.05). Statistical analysis was completed using SPSS 19.0 software (SPSS Inc., Chicago, IL).

## Results

Of the 127 patients, 27 were excluded from the study for not completing the questionnaire correctly. A total of 100 patients (52 women and 48 men) were included. Their mean age was 31 years (SD 10.9) range 18 to 69. The internal consistency of the items included in the questionnaire was very high (α = 0.94). The mean values of the variables are in the  Table 1.

**Table 1:**
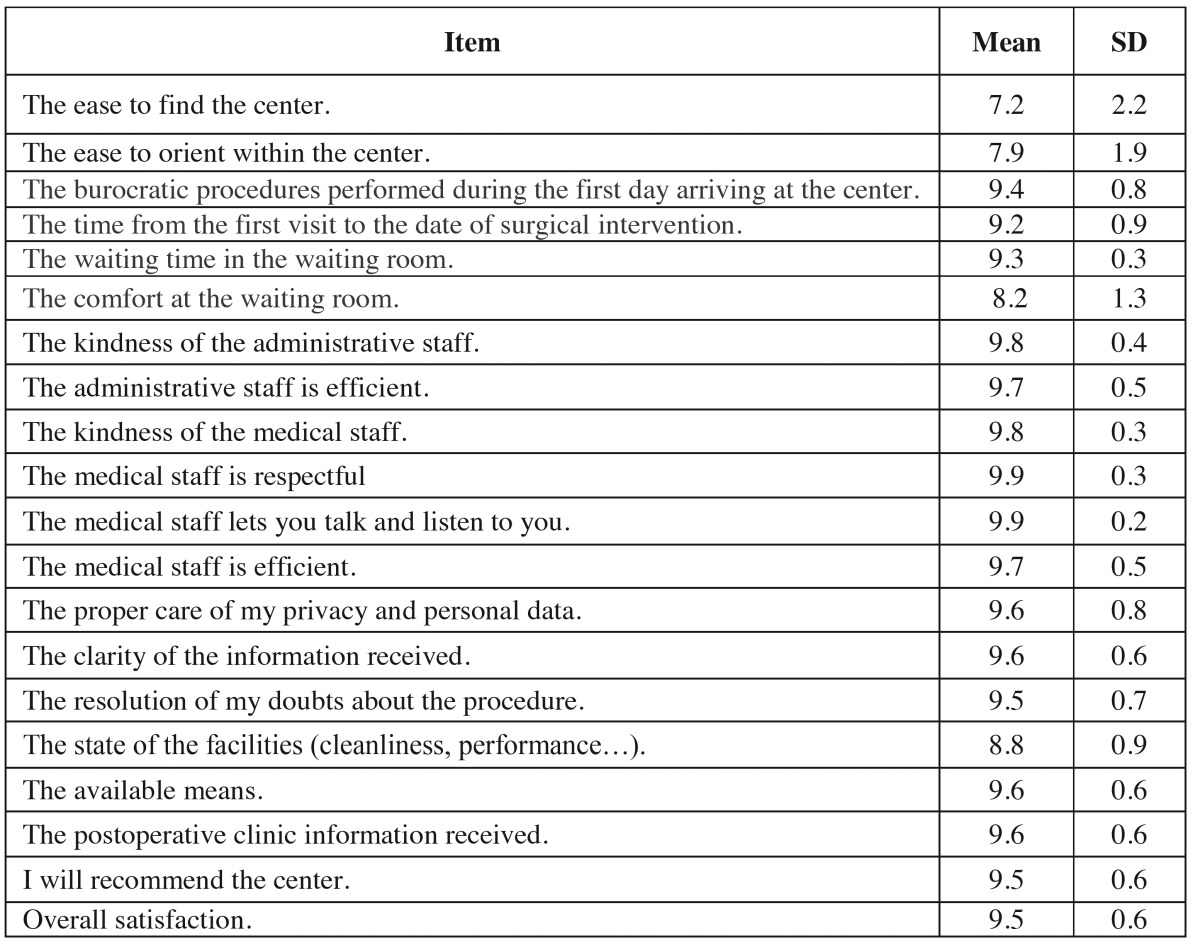
Mean and SD of the items included in the questionnaire.

- Overall satisfaction

The mean of the OS variable was 9.43 (SD=1.07) [95% CI=9.22-9.64]. The Spearman’s correlation coefficient showed a correlation higher than 0.7 between the OS and the kindness and efficiency of the management staff (0.727 and 0.711, respectively), the kindness, respectfulness and the time surgeon dedicated to talk and listen to the patient (0.767, 0.747 and 0.747 respectively), the efficiency of the surgeon (0.829), the clarity in the information received (0.807) and the state of facilities (0.808).

The multiple regression model showed a high goodness of fit after deleting the atypical values (R2=0.759). The statistically significant variables in the model were the practitioner’s efficacy (*p*<0.0001) and the clarity in the information received (*p*=0.02). The most important factor was the practitioner’s efficacy with a coefficient of 0.94, followed by the clarity of the information provided to the patient with 0.02. No statistically significant differences in the remaining variables were found.

The Cook’s distance was low in all the deleted atypical values (<0.2), therefore outliers did not influence the fit of the model.

- Recommendation of the centre

The mean of the ROC variable was 9.52 (SD=1.04) [95% CI=9.31-9.73]. A correlation higher than 0.7 was found between the ROC variable and the kindness and efficiency of the management staff (0.801 and 0.794), the kindness, respectfulness and efficiency of the medical staff (0.826, 0.819, 0.890), dedication (0.787), the clarity in the information received about the surgery and postoperative care (0.899 and 0.764, respectively), the resolution of doubts (0.840), the state of facilities (0.739) and the available means (0.893).

The multiple regression model showed a high goodness of fit after deleting the atypical values (R2=0.88). The statistically significant variables in the model were the practitioner’s efficacy (*p*<0.0001), the kindness of the administrative staff (*p*=0.005), the available means (*p*=0.005), the state of facilities (*p*=0.01), the comfort at the waiting room (*p*=0.01) and the clarity of the information received (*p*=0.02). The most important factor was the practitioner’s efficiency with a coefficient of 0.89, followed by the administrative staff efficiency (0.02), the available means (0.02), the state of facilities (0.019), the comfort at the waiting room (0.018) and the ease to find the center (0.015). No statistically significant differences in the remaining variables were found.

The Cook’s distance was moderate in three of the deleted atypical values (<0.35) and low in the two remaining (<0.02).

## Discussion

Some studies in the literature have evaluated the correlation between surgical skills, intraoperative outcomes and postoperative variables with the satisfaction of the patient (8,10) but did not take into account the importance of other factors not directly related to the surgical procedure. This study was designed to assess patient satisfaction after ITM surgery related to the centre (administrative and medical staff and facilities), not intraoperative surgical factors. The OS of the patients was high (9.43), similar to results reported in the literature (11). The results of this study are consistent with other published findings that the most important determinant of patient satisfaction after ITM surgery is the practitioner’s efficacy (8).

Emmert *et al*. (12) found that the second most frequently concern of the patients is the practitioner kindness (38%), just after the practitioner’s efficiency. Another important concern was dedication (35.9%). The relation of the patient with the professional is important in the overall satisfaction of the treatment (13). In the present study no statistically significant correlation was found between the kindnesses of the practitioner, dedication, and the OS or ROC, although mean values were high. However, the kindness of the staff was statistically significant.

Trust between patient and practitioner is based on confidentiality, if the patient feels that their personal data are not treated properly may lead to hide relevant information for diagnosis. In the present study, information about personal data care was explained to the patient who signed an informed consent. This may explain the high mean values (9.5), although not significant, reported. Some complications strongly affect patient quality of life, such as paresthesia of the alveolar, lingual or buccal nerve. These sensory disturbances may be irreversible, so it is important to inform the patient of the existence of such complications before surgery (14). No statistically significant correlation was found between the information provided to the patient, both preoperative and postoperative; and the OS or ROC. The patients are unable to recall in detail much of the information provided to them (14), to avoid this possible source of bias the questionnaire was delivered when the patient came back to remove the suture. Moreover, an excess of information does not always guarantee a greater understanding by the patient (15), may even be counterproductive and increase the level of patient anxiety (16), although van Wijk *et al*. (17) indicate that the patient is more satisfied when he gets as much information as possible. No statistically significant differences were found between gender or age to overall patient satisfaction, which is consistent with the literature (10).

## Conclusions

Patient satisfaction directly depends on the efficiency of the surgeon and the clarity of the clinical information received about the procedure. Appreciation of these predictive factors may help clinicians to provide optimal care and increasing patient satisfaction after impacted third molar surgery.
